# Association of *TERT* Polymorphisms with Clinical Outcome of Non-Small Cell Lung Cancer Patients

**DOI:** 10.1371/journal.pone.0129232

**Published:** 2015-05-28

**Authors:** Xueying Zhao, Shiming Wang, Junjie Wu, Xiaoying Li, Xun Wang, Zhiqiang Gao, Wenting Wu, Haijian Wang, Jiucun Wang, Ji Qian, Ke Ma, Hui Li, Baohui Han, Chunxue Bai, Qiang Li, Wenbin Liu, Daru Lu

**Affiliations:** 1 State Key Laboratory of Genetic Engineering and MOE Key Laboratory of Contemporary Anthropology, Institute of Genetics, School of Life Sciences, Fudan University, Shanghai, China; 2 Shanghai Key Laboratory of Crime Scene Evidence, Shanghai Research Institute of Criminal Science and Technology, Shanghai, China; 3 Department of Pneumology, Changhai Hospital of Shanghai, Second Military Medical University, Shanghai, China; 4 Department of Pulmonary Medicine, Zhongshan Hospital, Fudan University, Shanghai, China; 5 Department of Respiratory Disease, Shanghai Chest Hospital, Shanghai Jiaotong University, Shanghai, China; MOE Key Laboratory of Environment and Health, School of Public Health, Tongji Medical College, Huazhong University of Science and Technology, CHINA

## Abstract

*TERT* is of great importance in cancer initiation and progression. Many studies have demonstrated the *TERT* polymorphisms as risk factors for many cancer types, including lung cancer. However, the impacts of *TERT* variants on cancer progression and treatment efficacy have remained controversial. This study aimed to investigate the association of *TERT* polymorphisms with clinical outcome of advanced non-small cell lung cancer (NSCLC) patients receiving first-line platinum-based chemotherapy, including response rate, clinical benefit, progression-free survival (PFS), overall survival (OS), and grade 3 or 4 toxicity. Seven polymorphisms of *TERT* were assessed, and a total of 1004 inoperable advanced NSCLC patients treated with platinum-based chemotherapy were enrolled. It is exhibited that the variant heterozygote of rs4975605 showed significant association with a low rate of clinical benefit, and displayed a much stronger effect in never-smoking female subset, leading to the clinical benefit rate decreased from 82.9% (C/C genotype) to 56.4% (C/A genotype; adjusted OR, 3.58; *P*=1.40×10^-4^). It is also observed that the polymorphism rs2736109 showed significant correlation with PFS (log-rank *P*=0.023). In age > 58 subgroup, patients carrying the heterozygous genotype had a longer median PFS than those carrying the wild-type genotypes (*P*=0.002). The results from the current study, for the first time to our knowledge, provide suggestive evidence of an effect of *TERT* polymorphisms on disease progression variability among Chinese patients with platinum-treated advanced NSCLC.

## Introduction

Lung cancer remains the leading cause of cancer-related death worldwide [1]. Non-small cell lung cancer (NSCLC) accounts for about 85% of lung cancer cases, with the majority of patients presenting with advanced disease (stage IIIor IV), making surgical treatment impossible. Currently, platinum-based chemotherapy has been widely used as first-line treatment for advanced NSCLC, bringing modest benefits but also adverse effects, with the five-year survival rates less than 15% [2]. Clinical studies have suggested that the effects of platinum-based chemotherapy vary greatly among individuals [3,4]. Thus, the research of tumor and patient genetic profiles, in order to identify predictive biomarkers for better efficacy and minimal toxicity, may provide new opportunities for tailoring treatment.

Three genome-wide association study (GWAS) identified susceptibility variants in the telomerase reverse transcriptase (*TERT*) gene associated with lung cancer risk [5–7]. *TERT* encodes the catalytic subunit of the telomerase ribonucleoprotein complex, which catalyzes the addition of telomeric repeats at the ends of chromosomes, thus plays a crucial role in maintaining genome integrity, controlling cell proliferation, and regulating tissue homeostasis [8,9]. Telomerase is generally believed to be critical in cancer initiation and progression. Telomerase activation is a pivotal prerequisite for cell immortalization [10]. It lacks activity in most normal human cells with limited proliferative ratio, whereas it is activated in >90% of cancerous cells, making them grow continuously [11,12]. TERT expression is also increased in cancers, and prognostic significance was demonstrated in many cancer types [13–16]. Besides, functional and mechanistic studies have suggested that TERT acts as a direct transcriptional regulator of oncogenic signaling pathways that not only modulate its own levels but also control the induction of target genes critical for cell survival and cancer progression [8,17–20]. What is more, telomerase is reported to be the target of many kinds of anticancer agents, and both telomerase inhibition and telomere dysfunction have been demonstrated as a consequence of platinum treatment, suggesting the potential influence of TERT on treatment efficacy of platinum-based chemotherapy [21,22].

In molecular epidemiology studies, several *TERT* polymorphisms have been reported to be associated with the risk of many cancer types [10]. Only a few studies have paid attention to the associations between *TERT* variants and cancer progression, whereas the findings have remained controversial [13–16]. It is noteworthy that there are no reports concerning the correlation between *TERT* polymorphisms and clinical outcomes of cancer patients receiving platinum-based chemotherapy. In the current study, we investigated 7 single nucleotide polymorphisms (SNPs) in *TERT* gene and estimated the associations of the polymorphisms with treatment response, disease progression, prognosis, and adverse effects in 1004 advanced NSCLC patients receiving first-line platinum-based chemotherapy. The goal of this study was to explore the potential influence of *TERT* genetic variants on the treatment and progression of advanced NSCLC.

## Material and Methods

### Patients recruitment and follow-up

We recruited 1004 patients with inoperable and newly histologically diagnosed stage IIIA-IV non-small cell lung cancer in Shanghai, China, between March 2005 and January 2010. Patients enrolled in this study met the criteria defined previously [3,23]. Clinical information for all patients was recorded systematically at entry, including age at diagnosis, gender, smoking status, clinical stage, and tumor histology. Before chemotherapy, all the patients received evaluation including complete medical history, physical examination, and laboratory investigations. The medical record was available and reviewed by the oncologists. For smoking status, those who had smoked less than one cigarette per day and less than one year in their lifetime were defined as never smokers, otherwise they were considered ever smokers. Survival statistics were collected from several sources, including follow-up calls, Social Security Death Index, and inpatient and outpatient clinical medical records. Investigators were blinded to the genetic variant status of the patients. The research protocol was approved by the Ethical Review Committee of Fudan University and the participating hospitals, and written informed consent was obtained from each individual.

All the 1004 patients enrolled in the study were inoperable and were given first-line platinum-based chemotherapy (no prior surgery, radiotherapy, or concurrent chemoradiotherapy) as follows: cisplatin (75mg/m^2^), or carboplatin (AUC 5), both administered on day 1 every 3 weeks, in combination with navelbine (25 mg/m^2^), on days 1 and 8 every 3 weeks, or gemcitabine (1250 mg/m^2^), on days 1 and 8 every 3 weeks, or paclitaxel (175 mg/m^2^), on day 1 every 3 weeks, or docetaxel (75 mg/m^2^), on day 1 every 3 weeks. Few patients were given other platinum-based treatment. All chemotherapeutic drugs were administered intravenously, and all treatments were for two to six cycles.

The chemotherapy responses of the patients were assessed from the end of the first two cycles of treatment according to Response Evaluation Criteria in Solid Tumors (RECIST) guidelines version 1.0, which divided the responses into four categories: complete response (CR), partial response (PR), stable disease (SD), and progressive disease (PD). The response rate was defined as the percentage of patients with CR or PR, and clinical benefit represented those with CR, PR, or SD [24]. Progression-free survival (PFS) was calculated from the date of chemotherapy beginning to the date of disease progression or death (whichever occurred first). Overall survival (OS) was calculated from the date of chemotherapy beginning to the date of death.

The incidence of chemotherapy toxicity was evaluated twice weekly according to the National Cancer Institute Common Toxicity Criteria version 3.0. Toxicities included neutropenia, anemia, thrombocytopenia, nausea, and vomiting. Each of these was grouped into (i) grade 2 or less as mild toxicity, and (ii) grade 3 or 4 as severe toxicity. Grade 3 or 4 neutropenia, anemia, or thrombocytopenia composed the severe hematologic toxicity, and grade 3 or 4 nausea or vomiting composed the severe gastrointestinal toxicity [25]. No grade 5 toxicity (death) was observed.

### SNP Genotyping

The genotyping data on the *TERT* gene region (including 2kb upstream) from the Han Chinese in Beijing (CHB) population were acquired from the phase 2 HapMap SNP database (http://www.hapmap.org/), and tag-SNPs with a minor allele frequency (MAF) ≥ 0.05 and a correlation coefficient (*r* ^*2*^) threshold < 0.8 were selected using Haploview 4.1 (http://www.broadinstitute.org/haploview). Potentially functional SNPs published in the previous studies were also involved. Blood samples were collected from patients at the time of recruitment. Genomic DNA was prepared using QIAamp DNA Maxi Kit (Qiagen, Hilden, Germany). All selected SNPs were genotyped using a customized iSelect HD BeadChip (Illumina, San Diego, CA, USA), with the quality control criteria as follows: a genotyping call rate by SNP > 0.90, and a GenCall score > 0.2. A total of 7 SNPs passed the quality filters. Concordance between the genotyping replicates was above 99.9%.

### Statistical analysis

Statistical analyses were performed to investigate the relations between *TERT* SNPs and clinical outcomes (response rate, clinical benefit, PFS, OS, and severe toxicity) following platinum-based chemotherapy. Patient characteristics were tested against outcomes by chi-square tests (for dichotomous variables) or log-rank tests (for survival variables). The association between each genetic polymorphism and dichotomous outcomes were assessed by odd ratios (ORs) and 95% confidence intervals (CIs) using unconditional logistic regression model, with adjustment of patient characteristics (age, gender, Eastern Cooperative Oncology Group performance status, smoking status, TNM stage, histological type, and chemotherapy regimens) to show *P* < 0.1 in chi-square tests. The associations of genetic variants with survival were evaluated by log-rank test, and hazard ratios (HR) and 95% CI was calculated by Cox proportional hazards regression model, with adjustment of patient characteristics to yield *P* < 0.1 in log-rank tests. Kaplan-Meier method was used to plot survival curve. Genetic variants that yield *P* < 0.05 in multivariate analysis were selected for stratification analysis in characteristics subgroups, and *P* values for interaction were calculated to investigate the influences of genotypes on outcomes between characteristics subgroups. Pairwise linkage disequilibrium (LD) among polymorphisms were evaluated using *D’* and *r* ^*2*^, and haplotype block was defined by the Gabriel *et al* method using Haploview 4.1 [26]. The haplotype frequencies were calculated using Phase 2.0 program (version 2.0.2) [27]. All statistical analyses were performed using SPSS software (version 15.0), and a two-sided *P* value < 0.05 was considered statistically significant. For each SNP, Bonferroni correction was made for the *P* value by multiplying the number of SNPs tested in this study [3].

## Results

### Patient characteristics and clinical outcome

The patient characteristics and clinical outcomes of all 1004 patients enrolled in this study are shown in Table 1. All patients had received two to six cycles of first-line platinum-based chemotherapy. The correlations between variables were tested by chi-square tests or log-rank tests (data not shown). Of the subjects, the response rate to chemotherapy was 18.2%, and the clinical benefit was 80.7% (0.1% patients with CR, 18.1% patients with PR, 62.6% patients with SD, and 19.3% patients with PD). For survival analysis, by the time of final data collection (July 2012), the median follow-up time was 46.5 month, and death had occurred in 74.9% of enrolled patients. The median PFS was 9.1 months, and the median OS was 19.3 months. For toxicity analysis, 232 (23.9%) patients suffered from grade 3 or 4 hematologic toxicity, and 80 (8.3%) patients experienced grade 3 or 4 gastrointestinal toxicity. A few patients were not enrolled because of loss to follow-up. All 7 SNPs were in Hardy-Weinberg equilibrium (*P* > 0.05; Table 2).

**Table 1:** Patient Characteristics and Clinical Outcomes.

Patient Characteristics	Total N	N (%)
Total no. of patients	1004	
Median age (range)	1004	58 (26–82)
Age	1004	
≤58		518 (51.6)
>58		486 (48.4)
Gender	1004	
Male		706 (70.3)
Female		298 (29.7)
ECOG PS	990	
0–1		904 (91.3)
2		86 (8.7)
Smoking Status	1000	
Ever smokers		575 (57.5)
Never smokers		425 (42.5)
Never-Smoking Females		287 (28.7)
TNM stage	999	
IIIA		81 (8.1)
IIIB		293 (29.3)
IV		625 (62.6)
Histological type	1004	
Adenocarcinoma		632 (62.9)
Squamous cell		221 (22.0)
Adenosquamocarcinoma		20 (2.0)
Others^1^		131 (13.1)
Chemotherapy regimens	1004	
Platinum—navelbine		316 (31.5)
Cisplatin—navelbine		
Platinum—gemcitabine		239 (23.8)
Cisplatin—gemcitabine		
Platinum—paclitaxel		313 (31.2)
Carboplatin—paclitaxel		
Platinum—docetaxel		87 (8.7)
Other platinum combinations		49 (4.9)
Objective response	975	
CR		1 (0.1)
PR		176 (18.1)
SD		610 (62.6)
PD		188 (19.3)
Median time to outcomes (months)		
PFS	896	6.5
OS	972	16.0
Toxicity outcomes		
Grade 3 or 4 hematologic toxicity	969	232 (23.9)
Neutropenia	935	115 (12.3)
Anemia	944	29 (3.1)
Thrombocytopenia	950	34 (3.6)
Grade 3 or 4 gastrointestinal toxicity		
Nausea/Vomiting	964	80 (8.3)

Abbreviation: ECOG PS, Eastern Cooperative Oncology Group performance status; TNM, tumor-node-metastasis; CR, complete response; PR, partial response; SD, stable disease; PD, progressive disease; PFS, progression-free survival; OS, overall survival.

^1^ Other carcinomas include mixed cell or undifferentiated carcinoma.

**Table 2:** 7 genotyped SNPs of TERT gene.

NCBI SNP ID	Gene position	Base change	Genotyping rate (%)	*P* value for HWE	MAF
rs2736118	Intron 12	A>G	100.0	0.54	0.05
rs2075786	Intron 10	T>C	90.8	1.00	0.13
rs4975605	Intron 6	C>A	100.0	0.47	0.10
rs2736100	Intron 2	T>G	99.9	0.60	0.49
rs2853676	Intron 2	G>A	99.7	0.13	0.17
rs2736098	Exon 2	G>A	96.7	0.06	0.42
rs2736109	5’flank	G>A	100.0	0.53	0.36

Abbreviation: HWE, Hardy-Weinberg Equilibrium; MAF, minor allele frequency.

### Association of genetic variants with chemotherapy response

Associations between *TERT* polymorphisms and objective responses (response rate and clinical benefit) to first-line platinum-based chemotherapy were assessed using unconditional logistic regression model. We observed that the rate of clinical benefit was significantly lower in the variant heterozygote C/A of polymorphism rs4975605 than in the wild-type homozygote C/C (adjusted OR, 1.51; 95% CI, 1.01–2.25; *P* = 0.046; Table 3). Further stratification analyses between rs4975605 and clinical benefit were performed by patient characteristic subgroups of age, gender, smoking status, TNM stage, histological type, and treatment regimens. A dominant model was assumed because of the relatively small sample size in subgroup analysis. We observed that the variant rs4975605 showed the most significant correlation with clinical benefit in the never-smoking females subset (adjusted OR, 3.58; 95%CI, 1.86–6.90; *P* = 1.40×10^–4^; remaining significant after Bonferroni correction; Table 3), with a significant influence of interaction was exhibited (*P* = 0.003). Several significant associations between rs4975605 and clinical benefit were also observed in subgroups of ≤ 58 years old, female, never smokers, stage IV, histologic type of adenocarcinoma, and cisplatin-navelbine regimen.

**Table 3:** Association Between rs4975605 and Clinical Benefit.

	C/C		C/A		A/A		Heterozygositic		Homozygositic		Dominant	
Variables	No.^1^	%	No.^1^	%	No.^1^	%	OR (95%CI) ^2^	*P* ^2^	OR (95%CI) ^2^	*P* ^2^	OR (95%CI) ^2^	*P* ^2^
**Overall**	651/795	81.9	125/168	74.4	11/12	91.7	1.51(1.01–2.25)	0.046	0.41(0.05–3.26)	0.402	1.42(0.95–2.11)	0.085
**Age,years**												
** ≤58**	339/410	82.7	60/84	71.4	7/8	87.5	1.74(1.00–3.03)	0.049	0.66(0.08–5.52)	0.704	1.63(0.95–2.80)	0.077
** >58**	312/385	81.0	65/84	77.4	4/4	100.0	1.28(0.71–2.32)	0.412	/	/	1.20(0.67–2.17)	0.540
**Gender**												
** Male**	467/573	81.5	92/110	83.6	5/6	83.3	0.87(0.49–1.53)	0.615	0.99(0.11–8.73)	0.992	0.87(0.50–1.52)	0.624
** Female**	184/222	82.9	33/58	56.9	6/6	100.0	3.44(1.81–6.53)	1.57×10^–4^ ^3^	/	/	2.91(1.56–5.44)	0.001 ^3^
**Smoking status**												
** Ever smokers**	380/467	81.4	78/93	83.9	2/3	66.7	0.78(0.42–1.46)	0.434	2.83(0.24–32.94)	0.405	0.82(0.45–1.51)	0.525
** Never smokers**	271/328	82.6	46/74	62.2	9/9	100.0	2.91(1.66–5.11)	1.94×10^–4^ ^3^	/	/	2.43(1.40–4.21)	0.002 ^3^
** Never-Smoking Females**	179/216	82.9	31/55	56.4	6/6	100.0	3.58(1.86–6.90)	1.40×10^–4^ ^3^	/	/	3.01(1.59–5.70)	0.001 ^3^
**TNM stage**												
** IIIB**	188/231	81.4	39/49	79.6	3/4	75.0	1.05(0.47–2.36)	0.901	1.61(0.16–15.94)	0.686	1.09(0.50–2.37)	0.825
** IV**	405/496	81.7	70/103	68.0	8/8	100.0	2.08(1.28–3.38)	0.003 ^3^	/	/	1.86(1.15–2.99)	0.011
**Histological type**												
** Adenocarcinoma**	398/499	79.8	70/104	67.3	9/9	100.0	1.88(1.18–3.00)	0.008	/	/	1.68(1.06–2.65)	0.028
** Squamous cell**	151/175	86.3	33/40	82.5	2/2	100.0	0.90(0.32–2.56)	0.849	/	/	0.86(0.30–2.41)	0.767
**Chemotherapy regimens**												
** Cisplatin–navelbine**	214/250	85.6	35/51	68.6	1/1	100.0	2.65(1.28–5.49)	0.008	/	/	2.57(1.25–5.29)	0.011
** Cisplatin—gemcitabine**	149/179	83.2	33/45	73.3	6/7	85.7	1.79(0.79–4.05)	0.163	0.83(0.10–7.21)	0.865	1.62(0.75–3.53)	0.223
** Carboplatin—paclitaxel**	201/251	80.1	44/56	78.6	3/3	100.0	1.12(0.55–2.30)	0.753	/	/	1.05(0.51–2.13)	0.903

Abbreviation: OR, odd ratio; CI, confidence interval.

^1^ Numbers of patients show CR, PR, or SD for chemotherapy among all the patients in the same genotype group.

^2^ Data were calculated by unconditional logistic regression, with adjustment of patient characteristics with *P* < 0.1 in chi-square tests (For stratification analysis of histological type, adjusting covariate was ECOG PS; for all the other analysis, adjusting covariates were ECOG PS and histological type).

^3^ Significance remained after the Bonferroni correction.

### Association of genetic variants with survival

**Fig 1:**
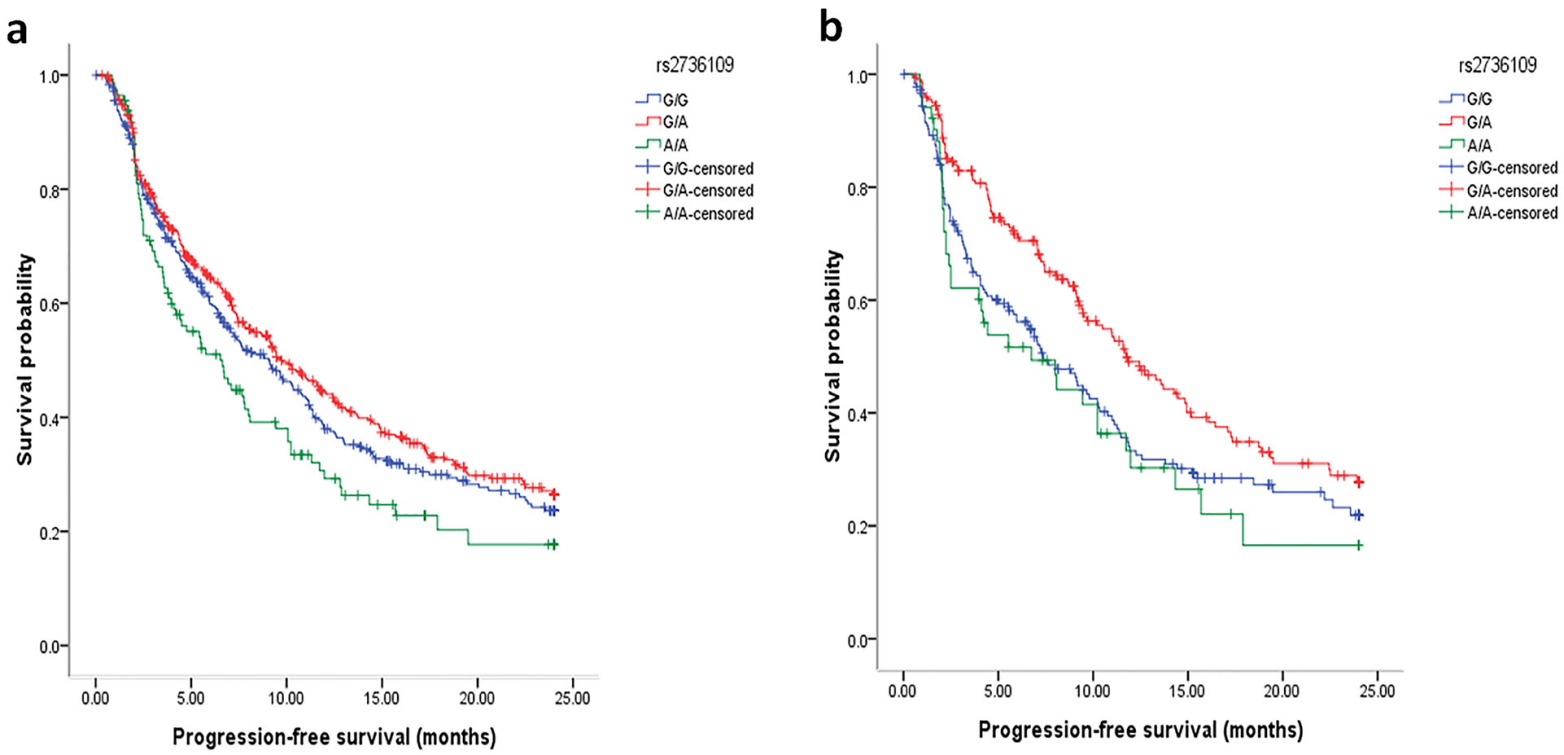
Kaplan-Meier curve of progression-free survival according to *TERT* rs2736109 polymorphism in (a) overall 1004 patients and (b) age > 58 supgroup. These plots were generated by the SPSS software.

Associations of *TERT* genetic variants with PFS and OS were evaluated using log-rank test and Cox proportional hazards regression model. It is observed that the polymorphism rs2736109 exhibited significant influence on PFS time of NSCLC patients after platinum-based treatment (log-rank *P* = 0.023). Stratification analyses by patient characteristic subgroups were performed and the most significant genetic model was assumed as the best fitting model. We observed that the median PFS time for patients with heterozygote G/A of rs2736109 was longer than those with the wild-type homozygous genotype in age > 58 subgroup (log-rank *P* = 0.007; adjusted HR, 0.65; 95%CI, 0.50–0.85; *P* = 0.002; remaining significant after Bonferroni correction; *P*_for interaction_ = 0.012; Table 4, Fig 1).

**Table 4:** Association Between rs2736109 and Progression—Free Survival.

		G/G	G/A	A/A		Heterozygositic		Homozygositic		Best Fitting Model		
Variables	No. ^1^	MST	MST	MST	Log-rank *P* ^2^	HR(95%CI) ^3^	*P* ^3^	HR(95%CI) ^3^	*P* ^3^	Log-rank*P* ^2^	HR(95%CI) ^3^	*P* ^3^
**Overall**	558/896	9.07	9.67	6.53	0.023	0.88(0.74–1.06)	0.171	1.23(0.95–1.60)	0.122	0.013	1.32(1.03–1.68)	0.026
**Age,years**												
** ≤58**	296/468	9.83	7.40	6.53	0.175	1.13(0.88–1.45)	0.344	1.34(0.93–1.93)	0.116	0.193	1.17(0.92–1.48)	0.199
** >58**	262/428	7.37	11.73	6.73	0.007 ^4^	0.65(0.50–0.85)	0.002 ^4^	1.13(0.77–1.67)	0.525	0.042	0.73(0.57–0.94)	0.014
**Gender**												
** Male**	392/629	8.80	10.97	6.53	0.015	0.80(0.64–0.99)	0.039	1.16(0.85–1.59)	0.340	0.038	1.31(0.98–1.76)	0.068
** Female**	166/267	9.47	7.17	3.53	0.271	1.19(0.85–1.68)	0.316	1.51(0.92–2.47)	0.103	0.161	1.36(0.87–2.12)	0.180
**Smoking status**												
** Ever smokers**	324/517	8.23	11.73	6.53	0.026	0.76(0.59–0.96)	0.022	1.08(0.77–1.52)	0.662	0.092	1.25(0.91–1.72)	0.177
** Never smokers**	233/378	9.17	7.33	5.53	0.174	1.11(0.83–1.48)	0.476	1.55(1.03–2.36)	0.038	0.070	1.46(1.00–2.14)	0.049
** Never-SmokingFemales**	160/259	9.20	7.17	3.53	0.273	1.21(0.85–1.72)	0.289	1.52(0.92–2.50)	0.099	0.158	1.36(0.87–2.13)	0.182
**TNM stage**												
** IIIB**	153/254	9.07	12.73	6.53	0.584	0.86(0.61–1.21)	0.388	1.38(0.81–2.34)	0.234	0.372	1.48(0.89–2.46)	0.134
** IV**	364/572	8.03	9.10	6.70	0.042	0.87(0.70–1.09)	0.236	1.23(0.89–1.71)	0.211	0.026	1.33(0.99–1.80)	0.062
**Histological type**												
** Adenocarcinoma**	362/573	9.63	9.50	6.73	0.144	0.92(0.73–1.15)	0.462	1.24(0.89–1.73)	0.197	0.064	1.30(0.96–1.77)	0.091
** Squamous cell**	117/191	7.60	12.40	4.50	0.053	0.77(0.51–1.17)	0.214	1.42(0.80–2.54)	0.233	0.050	1.64(0.95–2.81)	0.073
**Chemotherapy regimens**												
** Cisplatin–navelbine**	150/302	13.93	16.07	6.53	0.078	0.83(0.58–1.17)	0.281	1.47(0.88–2.46)	0.137	0.042	1.64(1.02–2.64)	0.041
** Cisplatin—gemcitabine**	144/210	10.83	9.13	7.67	0.776	1.08(0.75–1.55)	0.671	1.19(0.72–1.96)	0.496	0.618	1.14(0.72–1.80)	0.574
** Carboplatin—paclitaxel**	190/273	6.40	9.03	3.13	0.012	0.76(0.56–1.04)	0.089	1.46(0.94–2.28)	0.096	0.011	1.70(1.13–2.57)	0.011

Abbreviation: MST, median survival time (months); HR, hazard ratio; CI, confidence interval.

^1^ Numbers of patients show progression disease among all the patients in the same characteristics group.

^2^ Data were calculated by log-rank test.

^3^ Data were calculated by Cox proportional hazards regression model, with adjustment of patient characteristics with *P* < 0.1 in log-rank test (For stratification analysis of chemotherapy regimens, adjusting covariate was ECOG PS; for all the other analysis, adjusting covariates were ECOG PS and chemotherapy regimens).

^4^ Significance remained after the Bonferroni correction.

*TERT* has only one linkage disequilibrium (LD) block according to the reconstructed LD plot of 1004 patients (S1 Fig), which was defined by the method of Gabriel *et al*. The polymorphism rs2736109 was observed in high LD with rs2736098 (*D’* = 0.977; *r* ^*2*^ = 0.765). Further haplotype analyses were performed to investigate the relationships between *TERT* genetic variants and PFS. However, no significant association was observed between rs2736098-rs2736109 haplotypes and PFS, both in overall patients and in subgroup analysis (Data not shown).

### Association of genetic variants with grade 3 or 4 toxicity

Associations between each *TERT* SNP and each grade 3 or 4 toxicity were evaluated by unconditional logistic regression model. There was no statistically significant correlation between any polymorphisms and severe toxicity of platinum-based chemotherapy (Data not shown). The heterozygote C/A of rs4975605 showed a protective effect for severe neutropenia (adjusted OR, 0.58; 95%CI, 0.32–1.07, *P* = 0.081), and the heterozygous genotype of rs2736109 displayed a risk influence on severe gastrointestinal toxicity (adjusted OR, 1.68; 95%CI, 0.99–2.83, *P* = 0.054), but none of them reached the statistical significant level (Table 5).

**Table 5:** Association Between rs4975605, rs2736109 and Severe Toxicity.

	rs4975605					rs2736109				
Toxicity	Genotype	No.^1^	%	OR (95%CI) ^2^	*P* ^2^	Genotype	No. ^1^	%	OR (95%CI) ^2^	*P* ^2^
**Gastrointestinal Toxicity**	C/C	66/784	8.4	1		G/G	26/388	6.7	1	
	C/A	13/168	7.7	0.85(0.45–1.61)	0.615	G/A	45/454	9.9	1.68(0.99–2.83)	0.054
	A/A	1/12	8.3	0.86(0.11–7.06)	0.892	A/A	9/122	7.4	1.27(0.57–2.86)	0.560
**Hematologic Toxicity**	C/C	195/789	24.7	1		G/G	86/388	22.2	1	
	C/A	32/168	19.0	0.74(0.48–1.13)	0.165	G/A	124/459	27.0	1.29(0.94–1.78)	0.120
	A/A	5/12	41.7	2.50(0.77–8.12)	0.127	A/A	22/122	18.0	0.78(0.46–1.32)	0.348
**Neutropenia**	C/C	101/762	13.3	1		G/G	45/371	12.1	1	
	C/A	13/162	8.0	0.58(0.32–1.07)	0.081	G/A	60/446	13.5	1.11(0.73–1.68)	0.641
	A/A	1/11	9.1	0.93(0.12–7.57)	0.947	A/A	10/118	8.5	0.70(0.34–1.46)	0.344
**Anemia**	C/C	23/772	3.0	1		G/G	7/376	1.9	1	
	C/A	5/161	3.1	0.75(0.25–2.23)	0.601	G/A	18/448	4.0	1.90(0.77–4.71)	0.163
	A/A	1/11	9.1	2.85(0.32–25.84)	0.351	A/A	4/120	3.3	2.05(0.58–7.32)	0.267
**Thrombocytopenia**	C/C	30/777	3.9	1		G/G	13/377	3.4	1	
	C/A	3/162	1.9	0.38(0.11–1.29)	0.122	G/A	16/452	3.5	1.05(0.49–2.23)	0.908
	A/A	1/11	9.1	1.30(0.15–11.21)	0.814	A/A	5/121	4.1	1.12(0.38–3.27)	0.838

^1^ Numbers of patients experience grade 3 or 4 toxicity among all the patients in the same genotype group.

^2^ Data were calculated by unconditional logistic regression, with adjustment of patient characteristics with *P* < 0.1 in chi-square tests (For gastrointestinal toxicity, adjusting covariates were gender, smoking status, and chermotherapy regimens; for hematologic toxicity and neutropenia, chemotherapy regimens; for anemia, ECOG PS, Smoking status, TNM stage, and chemotherapy regimens; for thrombocytopenia, gender and chemotherapy regimens).

## Discussion

In this study, we systematically evaluated the associations of *TERT* polymorphisms with clinical outcome of advanced NSCLC patients receiving first-line platinum-based chemotherapy. We found that the variant heterozygote C/A of polymorphism rs4975605 exhibited significant association with a low rate of clinical benefit and, displayed a much stronger influence in the never-smoking female subgroup than the others, leading to the benefit rate that decreased from 82.9% (C/C genotype) to 56.4% (C/A genotype; *P* = 1.40×10^–4^). We also observed a significant correlation between the polymorphism rs2736109 and PFS, specially in age > 58 subgroup, with patients carrying the variant heterozygote G/A genotype having a longer median PFS than those carrying the wild-type homozygous genotypes (*P* = 0.002).

*TERT* is of great importance for genome integrity and plays a crucial role in cancer initiation and progression. Many molecular epidemiology studies have demonstrated genetic variations in the *TERT* gene as risk factors for many cancer types [5,10]. Mocellin *et al*. have summarized eighty-five studies of *TERT* polymorphisms enrolling 490901 subjects with a total of 24 tumor types, and have demonstrated the polymorphisms rs2736098, rs2736100, and rs2853676 significantly associated with risk of lung cancer [10]. What is more, several studies have reported that the polymorphism rs2736100 displayed significant correlation with lung cancer risk in never-smoking females [28–30]. Accumulating evidence has demonstrated that the molecular pathogenesis and treatment response of lung cancer differs by smoking status. Lung cancers arising in never smokers are suggested to have different molecular carcinogenic pathways and distinct profiles of oncogenic mutations; for example, lung cancer patients in never smokers are much more likely to carry mutations of epidermal growth factor receptor (*EGFR*) [31,32]. Functional and mechanistic studies have indicated that TERT plays an important role in regulating oncogenic signaling pathways and acts as a transcriptional regulator of EGFR expression, implying its potential role in the progression of lung cancers arising in never smokers [17,18,33]. In the current study, we did not observe significant association between rs2736100 and clinical outcomes of platinum-based chemotherapy, both in overall patients and in smoking status subgroups. However, the polymorphism rs4975605 showed a significant influence on clinical benefit after platinum-based treatment, and exhibited a much stronger effect in never-smoking female subgroup. This suggested that the variants rs4975605 displayed significant impact on the rate of progressive disease (PD) in advanced NSCLC patients following first-line platinum-based treatment. Futher analysis found that heterozygous rs4975605 exhibited significant influence on PFS in never-smoking female subgroup (adjusted HR, 1.50; 95%CI, 1.03–2.19; *P* = 0.037), suggesting its significant impact on advanced NSCLC progression.

In survival analyses, we observed the polymorphism rs2736109 significantly associated with PFS of advanced NSCLC patients receiving first-line platinum-based chemotherapy. The effect of the polymorphism was also apparent in subgroup of > 58 years old, male, ever smokers, never smokers, stage IV, histologic type of squamous cell, cisplatin-navelbine and carboplatin-paclitaxel regimens. The polymorphism rs2736109 is located in the putative promoter region of *TERT*. Interestingly, functional annotation indicates that rs2736109 is located in the binding site of GATA-2 transcription factor and the variant A allele creates a new GATA-1 binding site compared with the wild-type G allele according to TFSEARCH prediction (http://www.cbrc.jp/research/db/TFSEARCH.html), implying its potential impact on transcription factor binding activity and TERT expression. Further functional and mechanistic studies will be required to clarify the real role of this genetic variant. In this study, we did not observe any association between *TERT* polymorphisms and OS. OS as an endpoint of cancer treatment is clinically meaningful and objectively assessed, with limitations of confounding effects of post-protocol events, for example, subsequent therapies. Since we focused on first-line treatment in the current study, we believed PFS, with greater statistical power and a lower likelihood of confounding by subsequent therapies, is more suitable for our research than OS [34–36].

We performed the association analyses between *TERT* polymorphisms and grade 3 or 4 toxicity in advanced NSCLC patients during first-line platinum-based chemotherapy, however, no statistical significant association was observed. Platinum compounds can form both interstrand and intrastrand DNA adducts that result in DNA instability and cell death, which are responsible for the cytotoxicity of the platinum agents in both tumor cells and normal cells [3]. Telomerase is believed to be inhibited in most normal human cells, only remaining activity in some tissues, such as stem cell populations, male germ cells, and activated lymphocytes [9]. Interestingly, in the current study, we observed that the heterozygous rs4975605, which showed a significant influence on low benefit rate, displayed a protective effect for the incidence of grade 3 or 4 neutropenia (*P* = 0.081); similarly, the heterozygote of rs2736109, which showed significant correlation with long median PFS, exhibited a risk impact on grade 3 or 4 gastrointestinal toxicity occurrence (*P* = 0.054), although none of them reached the statistical significant level. These suggest that the *TERT* variants show consistent response to chemotherapy agents in tumor cells and normal cells.

In the current pharmacogenetic study, all 1004 advanced NSCLC patients enrolled are Han Chinese and were treated in three medical centers with exact same criteria for patient recruitment and information collection, limiting the potential confounding effects of clinical heterogeneity. The sample size of 1004 patients is large, may be one of the largest groups reported to date. However, when focused on subgroup analyses, the sample size in some subgroups, such as each single regimen, were still small. Detailed research in single regimen subsets require studies with larger sample sizes in the future. The most significant findings in this study, rs4975605 with clinical benefit and rs2736109 with PFS, all appeared in heterozygotes, which may also be a result of limited sample size. Because of the relatively small number of homozygous variants (for example, rs4975605, MAF = 0.1), especially in subgroups, there may be insufficient statistical power to detect the real effect of the homozygotes. Alternatively, this effect might be more likely to be subject to selection bias than that of the heterozygotes, which are often present in a larger number of subjects [37]. In addition, it is also possible that loss of heterozygosity (LOH) in this gene region [38], or the variant plays a role in an overdominant model, with the heterozygotes functioning in an unclear mode. These need to be examined in further studies.

To the best of our knowledge, this is the first study demonstrating significant associations between *TERT* polymorphisms and platinum-based chemotherapy outcomes in Chinese patients with advanced NSCLC. Our findings, although obtained from a retrospective study, suggest that *TERT* variants may play an important role in interindividual differences in disease progression of advanced NSCLC patients following platinum-based chemotherapy. Our results may be an interesting start point for future investigation and, further studies will be necessary to validate our findings.

## Supporting Information

S1 Fig Graphical representation of the SNP locations and block structure of *TERT* in our population samples. Pairwise linkage disequilibrium relationships between the *TERT* polymorphisms were reported using the correlation coefficient *r* ^*2*^.
(TIF)
