# Supplementary figures and images for: Association of TERT Polymorphisms with Clinical Outcome of Non-Small Cell Lung Cancer Patients
Source: PLoS One. 2015 May 28;10(5):e0129232. doi: 10.1371/journal.pone.0129232 (PMC4447454; doi:10.1371/journal.pone.0129232)

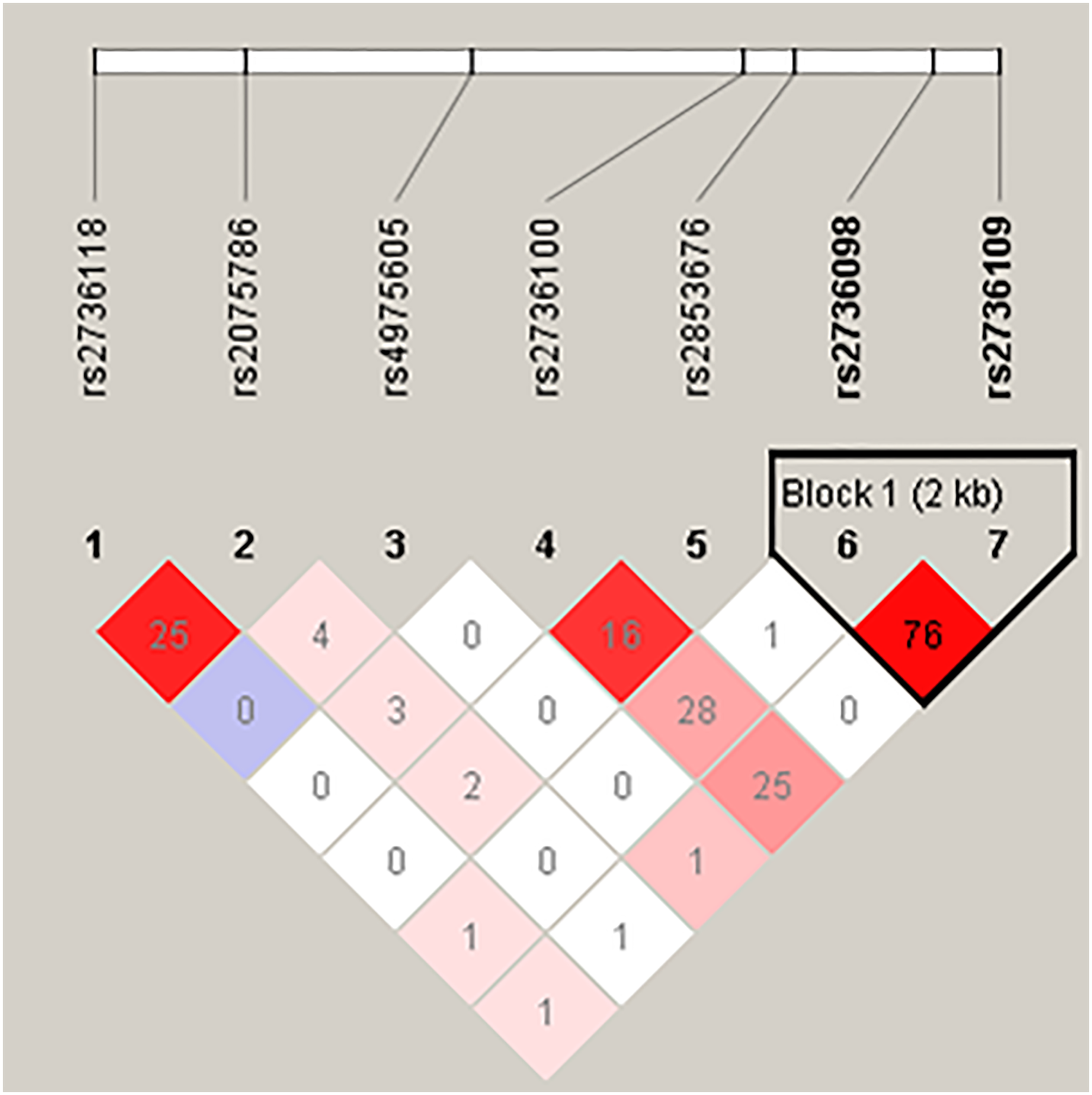

Supplement: S1 Fig — Pairwise linkage disequilibrium relationships between the TERT polymorphisms were reported using the correlation coefficient r 2. (TIF) [file pone.0129232.s001.tif]
